# Genome-wide association study and candidate gene analysis of rice cadmium accumulation in grain in a diverse rice collection

**DOI:** 10.1186/s12284-018-0254-x

**Published:** 2018-11-21

**Authors:** Junliang Zhao, Wu Yang, Shaohong Zhang, Tifeng Yang, Qin Liu, Jingfang Dong, Hua Fu, Xingxue Mao, Bin Liu

**Affiliations:** 1 Guangdong Key Laboratory of New Technology in Rice Breeding, Guangzhou, 510640 China; 2 Rice Research Institute, Guangdong Academy of Agricultural Sciences, Guangzhou, 510640 China

**Keywords:** Rice (*Oryza sativa* L.), Cadmium accumulation, Quantitative trait locus (QTL), Genome-wide association study (GWAS)

## Abstract

**Background:**

Cadmium (Cd) accumulation in rice followed by transfer to the food chain causes severe health problems in humans. Breeding of low Cd accumulation varieties is one of the most economical ways to solve the problem. However, information on the identity of rice germplasm with low Cd accumulation is limited, particularly in *indica*, and the genetic basis of Cd accumulation in rice is not well understood.

**Results:**

Screening of 312 diverse rice accessions revealed that the grain Cd concentrations of these rice accessions ranged from 0.12 to 1.23 mg/kg, with 24 accessions less than 0.20 mg/kg. Three of the 24 accessions belong to *indica*. *Japonica* accumulated significantly less Cd than *indica* (*p* < 0.001), while tropical *japonica* accumulated significantly less Cd than temperate *japonica* (*p* < 0.01). GWAS in all accessions identified 14 QTLs for Cd accumulation, with 7 identified in *indica* and 7 identified in *japonica* subpopulations. No common QTL was identified between *indica* and *japonica*. The previously identified genes (*OsHMA3*, *OsNRAMP1,* and *OsNRAMP5*) from *japonica* were colocalized with QTLs identified in *japonica* instead of *indica*. Expression analysis of *OsNRAMP2*, the candidate gene of the novel QTL (*qCd3–2*) identified in the present study, demonstrated that *OsNRAMP2* was mainly induced in the shoots of high Cd accumulation accessions after Cd treatment. Four amino acid differences were found in the open reading frame of *OsNRAMP2* between high and low Cd accumulation accessions. The allele from low Cd accumulation accessions significantly increased the Cd sensitivity and accumulation in yeast. Subcellular localization analysis demonstrated OsNRAMP2 expressed in the tonoplast of rice protoplast.

**Conclusion:**

The results suggest that grain Cd concentrations are significantly different among subgroups, with Cd concentrations decreasing from *indica* to temperate *japonica* to tropical *japonica*. However, considerable variations exist within subgroups. The fact that no common QTL was identified between *indica* and *japonica* implies that there is a different genetic basis for determining Cd accumulation between *indica* and *japonica*, or that some QTLs for Cd accumulation in rice are subspecies-specific. Through further integrated analysis, it is speculated that *OsNRAMP2* could be a novel functional gene associated with Cd accumulation in rice.

**Electronic supplementary material:**

The online version of this article (10.1186/s12284-018-0254-x) contains supplementary material, which is available to authorized users.

## Background

Cadmium (Cd) is one of the most mobile and toxic heavy metals. Due to rapid industrialization and environmental pollution, Cd pollution has become a major problem in paddy fields. It has been reported that approximately 20 million hectares of cultivated lands in China, or 20% of the total, were contaminated by Cd (Hu et al. 2016). Cd can be readily absorbed by the roots of crops and transferred to aboveground organs, then enter the human body through the food chain (Li et al. 2017). Rice is a staple food for nearly half of the world’s population, and compared to other cereal crops, rice tends to accumulate more Cd (Hu et al. 2016). A recent survey showed that 10% of rice grain with Cd exceeded the allowable concentration of 0.2 mg/kg as stipulated by the National Food Hygiene Standard of China (Xie et al. 2017). As rice is the primary source of dietary Cd intake (Clemens and Ma 2016; Uraguchi and Fujiwara 2013), Cd contamination in rice is becoming a severe public health problem.

Several techniques have been proposed to minimize Cd contamination in rice, such as phytoremediation and soil washing. Murakami et al. (2009) demonstrated that phytoextraction with the *indica* rice Chokoukoku, grown for 2 years without irrigation after drainage, removed 883 g Cd ha^− 1^, reducing the total soil Cd content by 38%. Makino et al. (2008) tested the feasibility of extracting Cd from the soil with iron, manganese, and zinc salts. Their results revealed that manganese, zinc, and iron salts extracted 4–41%, 8–44%, and 24–66% of the total Cd, respectively. Although these techniques are effective, they are time-consuming, expensive, or impractical in some situations. Many studies have revealed that Cd accumulation in rice varied considerably among cultivars and suggested that the breeding of low Cd-accumulating rice cultivars is a feasible strategy (Arao and Ae 2003; Cao et al. 2014; Yu et al. 2006). To date, breeding and use of rice cultivars with low Cd concentration in grain has been considered a promising solution to reducing Cd contamination in rice.

Obtaining rice germplasm with low Cd accumulation and identifying the genes responsible for Cd intake and transport are the prerequisites for the effective breeding of low-Cd-accumulation rice. In the past decade, much effort has been dedicated to the identification of rice germplasm with low Cd accumulation and of QTLs/genes controlling Cd accumulation (Ishikawa et al. 2005; Miyadate et al. 2011; Shimo et al. 2011). Ishikawa et al. (2005) conducted the first QTL analysis of Cd accumulation in rice, using 39 chromosome segment substitution lines derived from “Kasalath” and “Koshihikari”. They identified QTLs controlling the Cd concentration on chromosomes 3, 6, and 8 in brown rice. Since then, many QTLs for Cd accumulation in rice have been identified and mapped (Ueno et al. 2009; Xue et al. 2009; Norton et al. 2010; Ishikawa et al. 2010; Abe et al. 2013). Several genes have been cloned, and their functions have been confirmed regarding intake and transport of Cd in rice (Clemens and Ma 2016; Uraguchi and Fujiwara 2013). The P_1B_-type Heavy Metal ATPase 3 (*OsHMA3*) is the first cloned gene responsible for Cd accumulation in rice (Miyadate et al. 2011; Ueno et al. 2010). *OsHMA3* is expressed in the tonoplast of root cells, and limits translocation of Cd from the roots to the aboveground tissues by selectively sequestrating Cd into the root vacuoles (Miyadate et al. 2011; Ueno et al. 2010). Some *indica* and temperate *japonica* cultivars contain the non-functional allele of *OsHMA3*, which is related to higher Cd accumulation in these cultivars (Ueno et al. 2010; Yan et al. 2016). *OsHMA2* is another HMA family gene confirmed to be responsible for Cd accumulation in rice. Studies on OsHMA2 suggest that it plays a role in Cd loading to the xylem and participates in root-to-shoot translocation of Cd (Satoh-Nagasawa et al. 2012; Takahashi et al. 2012). Another study by Yamaji et.al (2013) found that OsHMA2 also plays an important role in preferential distribution of Cd through the phloem to the developing tissues. Additionally, *OsNRAMP1* and *OsNRAMP5*, belonging to the natural resistance-associated macrophage protein (NRAMP) family, were also identified as Cd transporters in rice (Sasaki et al. 2012; Takahashi et al. 2011). OsNRAMP1 participates in cellular Cd uptake and Cd transport within plants, and the higher expression of OsNRAMP1 in the roots could lead to an increase in Cd accumulation in the shoots (Takahashi et al. 2011). OsNRAMP5 is a plasma membrane-localized protein polarly localized at the distal side of both exodermis and endodermis cells. Functional analysis revealed that OsNRAMP5 is responsible for the transport of Mn and Cd from the external solution to root cells (Ishikawa et al. 2012; Sasaki et al. 2012). Despite significant progress being made in the identification of rice germplasm and associated QTLs/genes with low Cd accumulation, identification of both the *indica* germplasm with low accumulation and functional genes associated with low Cd accumulation from *indica* are limited.

Fortunately, rice germplasm is very rich, providing a good condition in searching for rice cultivars with low Cd accumulation. Genome-wide association study (GWAS) has emerged as a powerful approach for identifying the genes underlying complex traits at an unprecedented rate (Huang et al. 2010). This approach was first applied to the genetic analysis of human diseases (Hirschhorn and Daly 2005), and then used extensively for the genetic dissection of complex traits in plants (Li et al. 2016; Weng et al. 2011; Yang et al. 2014). With the rapid development in next-generation sequencing technology, GWAS with high-density molecular markers and more diverse resources is possible. Recently, McCouch et al. (2016) established an open-access resources for GWAS in rice, which included a collection consisting of 1568 diverse inbred rice varieties and their genotypes determined by 700,000 single-nucleotide polymorphisms (SNPs), providing a good resource for the genetic dissection of complex traits in rice through GWAS.

To identify rice germplasms with low Cd accumulation and the responsible genes, 312 diverse rice accessions, selected from the 1568 rice accessions (McCouch et al. 2016), were subjected to evaluation of Cd accumulation; GWAS was performed to map QTLs associated with low Cd accumulation in the present study. Twenty-four rice accessions were identified, including three *indica* varieties, with grain Cd accumulation less than 0.2 mg/kg. In total, 14, 7, and 7 QTLs for low Cd accumulation in grain were identified by GWAS, using all 312 rice accessions, in the *indica* subpopulation, and in the *japonica* subpopulation, respectively. The previously identified functional genes, including *OsHMA3*, *OsNRAMP1*, *OsNRAMP5*, and *OsLCD*, co-localized with the QTLs identified in the present study, suggesting the reliability of the GWAS results. Through differential expression analysis, genomic sequence analysis, yeast expression experiments, and subcellular localization analysis, *Os03g0208500,* a member of the rice NRAMP gene family, was identified as the possible functional gene of a novel QTL *(qCd3–2)* identified in the present study. The results provide a good basis for cloning of the gene and molecular breeding for low Cd accumulation in rice.

## Results

### Phylogenetic analysis of 312 rice accessions

A total of 312 rice accessions, which are from 53 countries worldwide, were selected from 1568 rice accessions (McCouch et al. 2016) according to their diversity of genotypes, origins, and subpopulations. Phylogenetic tree (Fig. S1) of the selected 312 rice accessions was constructed using SNP data from the previous study (McCouch et al. 2016). The phylogenetic analysis showed that 312 rice accessions could be divided into two subpopulations, namely *indica* population and *japonica* population, according to their genetic similarities. The *indica* population consists of 213 rice accessions, while the *japonica* population consists of 99 rice accessions. The *japonica* population can be further divided into two groups (temperate *japonica* group and tropical *japonica* group) according to the phylogenetic analysis and information provided by the previous study (McCouch et al. 2016).

### Variations in cd accumulation in grain among 312 rice accessions

**Fig. 1:**
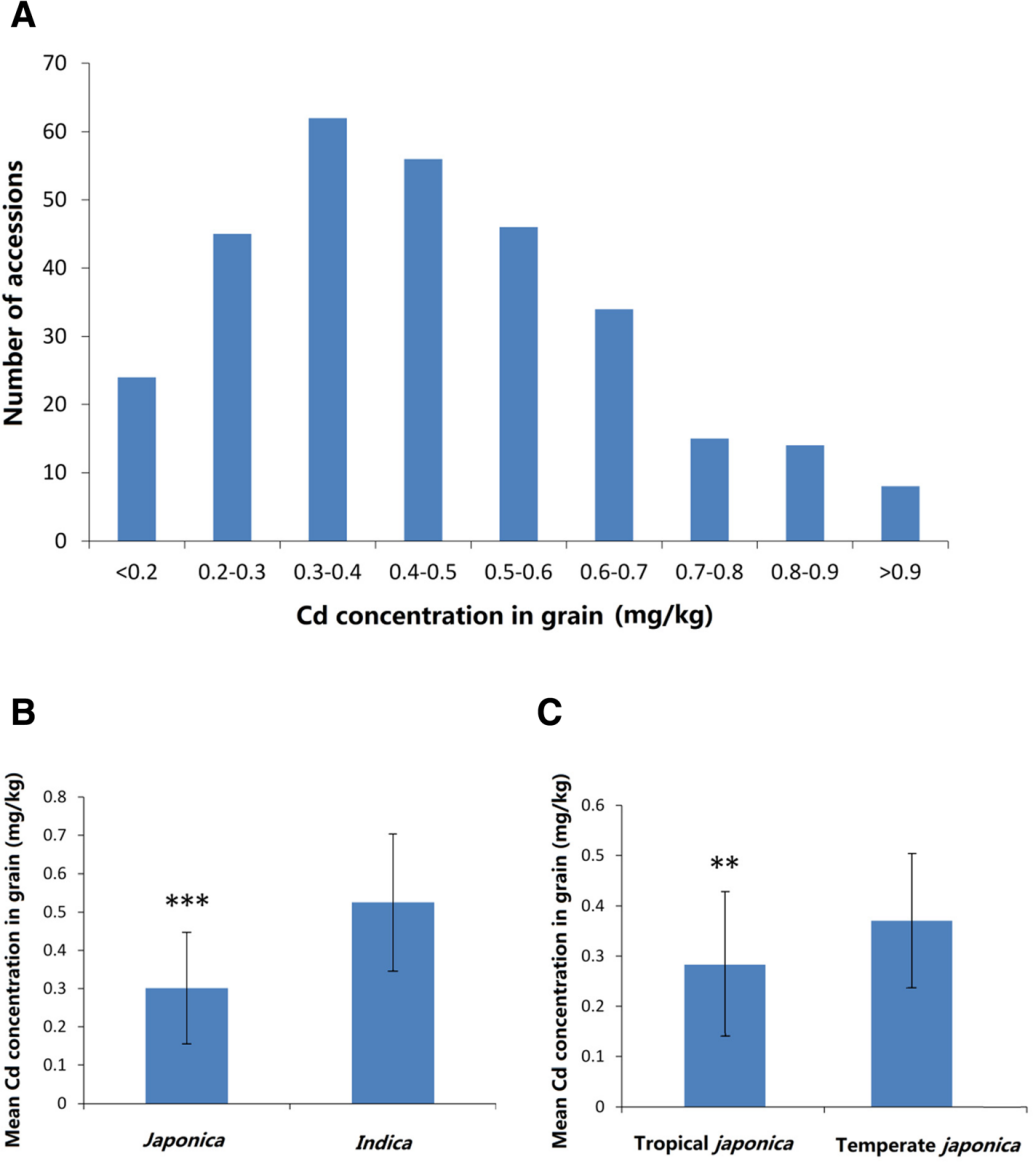
Distribution of Cd concentration in grain in 312 rice accessions and comparisons of Cd concentration in grain between subspecies and between subgroups. **a**, Distribution of Cd concentration in grain of 312 rice accessions. **b**, Comparison of Cd concentration in grain between *indica* and *japonica* species. **c**, Comparison of Cd concentration in grain between Temperate and Tropical *japonica*. *** and ** represent significant difference at *p* < 0.001,and *p* < 0.01 in *t*-test

Substantial variation and normal distribution in grain Cd accumulation were observed in 312 rice accessions (Fig. 1A). The *indica* cultivar “BOL ZO” from Korea Republic had the lowest Cd accumulation in grain (0.12 mg/kg), while the *indica* cultivar “Jariyu” from India had the highest Cd accumulation in grain (1.23 mg/kg). In total, 24 rice accessions had Cd accumulation in grain lower than 0.20 mg/kg (Table 1). Three out of those 24 rice accessions belong to *indica* and the others belong to tropical *japonica*. The comparisons in grain Cd accumulation of subspecies and subgroups revealed that the mean grain Cd accumulation of *indica* was significantly higher than that of *japonica* (*p* < 0.001) (Fig. 1B), while the mean grain Cd accumulation of temperate *japonica* was markedly higher than that of tropical *japonica* (*p* < 0.01) (Fig. 1C).

**Table 1:** The rice accessions with Cd concentration lower than 0.2 mg/kg in grain

Accession name	Subpopulation	Origin	Cd concentration in grain (mg/kg)
BOL ZO	*Indica*	Korea Rep	0.12
IAC 164	Tropical *Japonica*	Brazil	0.13
CHERIVIRUPPU	*Indica*	India	0.14
LEVANTE HOMEM	Tropical *Japonica*	Brazil	0.15
PACHOLINHA	Tropical *Japonica*	Brazil	0.15
ARAGUAIA	Tropical *Japonica*	Brazil	0.15
KWADWO AMOA	Tropical *Japonica*	Ghana	0.15
GBANTE	Tropical *Japonica*	Ivory Coast	0.16
MUT IAC 25–44-807	Tropical *Japonica*	Guyana	0.16
TOANG	Tropical *Japonica*	Indonesia	0.16
KETAN MERAH	Tropical *Japonica*	Indonesia	0.16
PATO DE GALLINAZO	Tropical *Japonica*	Australia	0.16
BOTRA FOTSY	Tropical *Japonica*	Madagascar	0.16
VARY MAINTY	Tropical *Japonica*	Madagascar	0.16
VARY SOMOTRA SIHANAKA	Tropical *Japonica*	Madagascar	0.17
MANGAVAVA FOTSILANSTSIKA	Tropical *Japonica*	Madagascar	0.17
BAKAW	Tropical *Japonica*	Philippines	0.18
HONDURAS	Tropical *Japonica*	Spain	0.18
IRAT 364	Tropical *Japonica*	Nicaragua	0.18
ARC 18294	*Indica*	India	0.19
RXAR RGUE:	Tropical *Japonica*	United States	0.19
WAB 56–125	Tropical *Japonica*	Ivory Coast	0.19
1–52-6	Tropical *Japonica*	Brazil	0.19
BOMALASANG	Tropical *Japonica*	Philippines	0.19

### Mapping of QTLs for cd accumulation in grain by GWAS

**Fig. 2:**
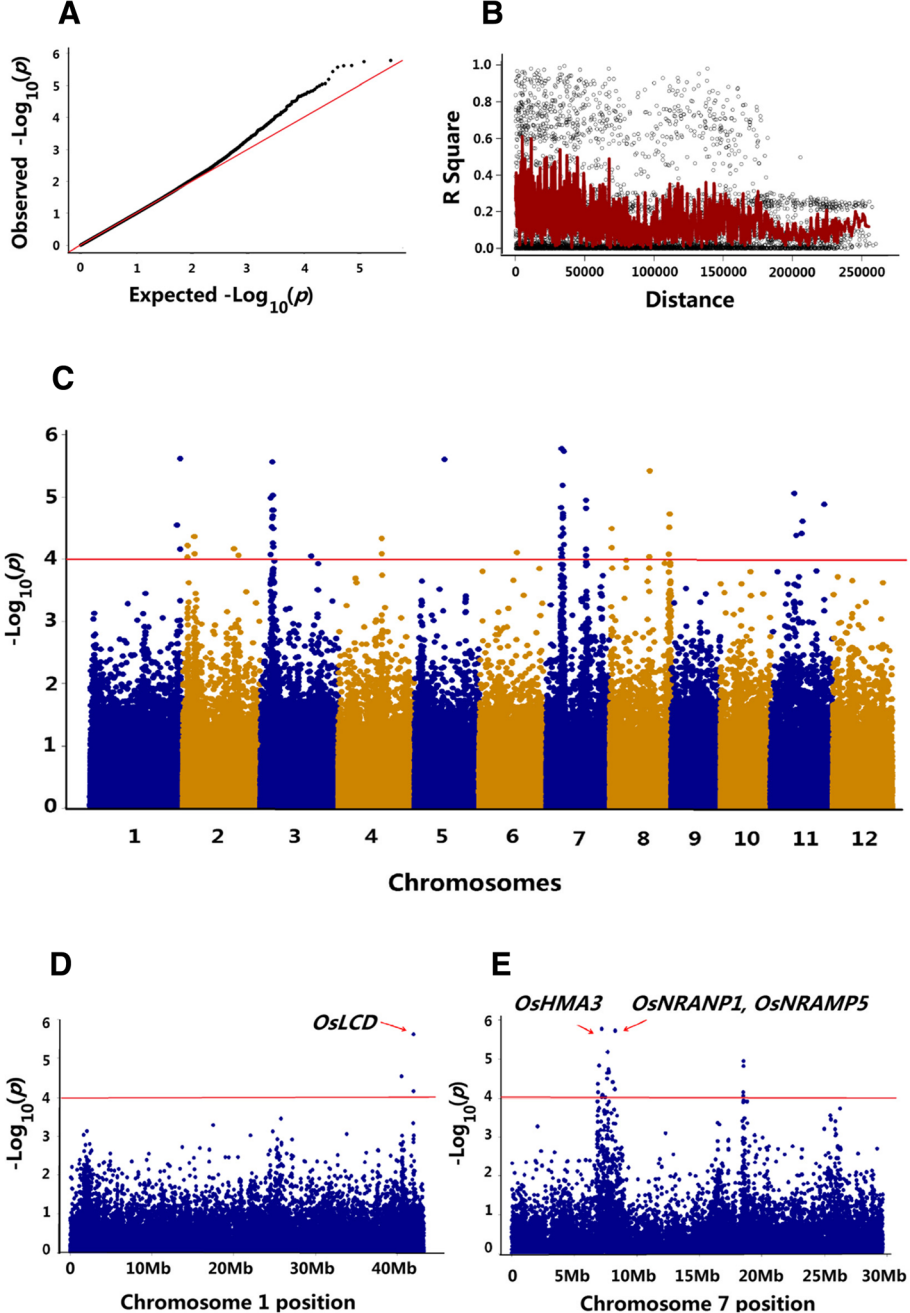
GWAS of grain Cd accumulation using the 312 rice accessions. **a**, QQ-plot for GWAS of Cd concentration in grain. **b**, LD decay of the whole population. **c**, Manhattan plots of GWAS of grain Cd accumulation in 12 chromosomes. **d**, Colocalization of *OsLCD* with *qCd-1*. **e**, Co-localization of *OsHMA3*, *OsNRAMP1* and *OsNRAMP5* with *qCd3–1* and *qCd3–2*

Based on the criteria of having less than 15% missing data and minor allele frequency (MAF) larger than 5% in the selected population, 183,884 SNPs were selected for GWAS from the SNP dataset in the present study (McCouch et al. 2016). The QQ plot is shown in Fig. 2A. According to the LD decay rate of 312 rice accessions, a significant LD decay was observed after 200 kb (Fig. 2B). Therefore, a region was considered as one QTL where it had more than two SNPs with *P* < 0.0001 within a 200-kb genomic window. The Manhattan plot of the GWAS results is shown in Fig. 2C. In total, 14 QTLs with 49 SNPs were significantly associated with grain Cd accumulation in the 312 rice accessions (Table 2). These QTLs (designate as *qCd* hereafter) distributed on chromosomes 1, 2, 3, 4, 7, 8 and 11, with detailed information of the 14 QTLs listed in Table 2 of this report. It is noted that three QTLs identified in the present study co-localized with the four previously cloned genes associated with Cd accumulation in rice (*OsLCD, OsHMA3, OsNRAMP1, OsNRAMP5*) (Miyadate et al. 2011; Sasaki et al. 2012; Shimo et al. 2011; Takahashi et al. 2011) (Fig. 2 D and 2E) (Table 2).

**Table 2:** QTL associated with Cd accumulation identified by GWAS using different populations

QTLs	Chromosome	Linked SNP position ^a^	*P*-value	Phenotype contribution (%)	Candidate gene
Composite population					
*qCd1*	1	41,982,531	2.43E-06	4.66	*OsLCD*
*qCd2–1*	2	2,060,980	6.01E-05	2.40	
*qCd2–2*	2	5,523,027	4.38E-05	3.47	
*qCd2–3*	2	23,732,572	6.88E-05	2.71	
*qCd3–1*	3	4,848,498	1.04E-05	4.06	
*qCd3–2*	3	5,635,117	2.78E-06	4.61	
*qCd4*	4	20,074,216	4.67E-05	2.53	
*qCd7–1*	7	7,186,204	1.69E-06	4.82	*OsHMA3*
*qCd7–2*	7	8,263,482	1.86E-06	4.78	*OsNRAMP1*, *OsNRAMP5*
*qCd7–3*	7	18,553,958	1.14E-05	4.02	
*qCd8–1*	8	902,085	3.25E-05	3.01	
*qCd8–2*	8	18,365,933	3.82E-06	4.48	
*qCd8–3*	8	27,463,194	3.12E-05	2.60	
*qCd11*	11	14,891,373	2.48E-05		
Indica					
*qCd_ind_1*	1	41,982,531	8.89E-05	6.85	*OsLCD*
*qCd_ind_2–1*	2	2,060,980	7.66E-04	5.00	
*qCd_ind_2–2*	2	4,986,931	2.27E-04	6.04	
*qCd_ind_3*	3	27,195,551	3.06E-04	5.78	
*qCd_ind_4*	4	20,368,258	1.34E-04	5.93	
*qCd_ind_5*	5	23,588,056	4.53E-04	5.45	
*qCd_ind_8*	8	899,377	1.93E-05	8.20	
Japonica					
*qCd_jap-2*	2	23,666,135	5.23E-05	12.74	
*qCd_jap-3*	3	5,507,369	3.81E-04	9.63	
*qCd_jap-7-1*	7	7,451,789	3.52E-04	9.75	*OsHMA3*
*qCd_jap-7-2*	7	8,467,983	1.56E-04	11.01	*OsNRAMP1*, *OsNRAMP5*
*qCd_jap-7-3*	7	24,677,317	3.71E-05	13.29	
*qCd_jap-7-4*	7	27,037,840	6.24E-04	8.88	
*qCd_jap-11*	11	25,263,712	4.09E-04	9.52	

^a^Position of SNP is base on rice reference sequence MSU V 7.0. (Kawahara et al. 2013)

**Fig. 3:**
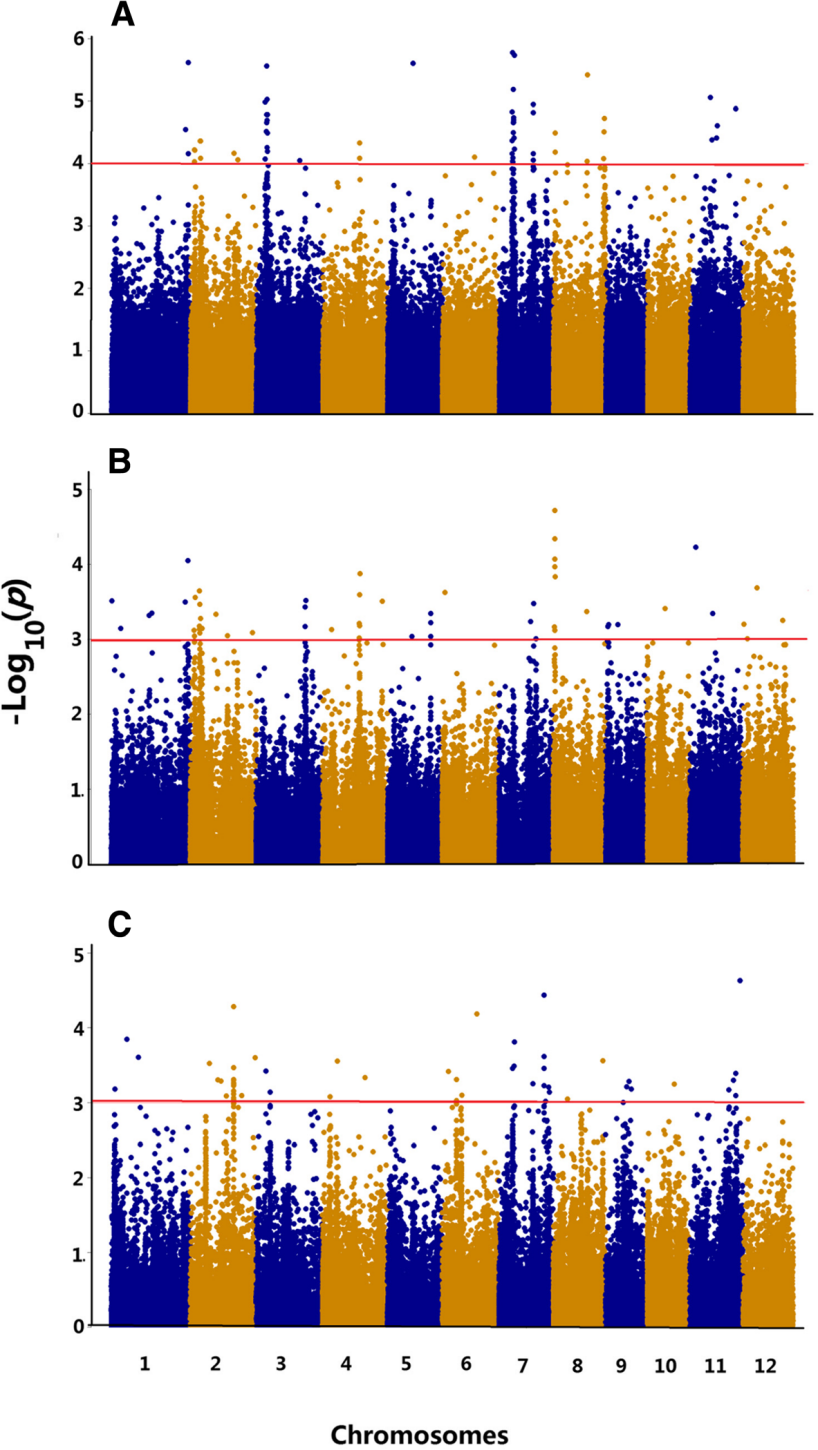
Manhattan plots of GWAS of grain Cd accumulation using different populations. **a**, Composite population. **b**, *Indica* subpopulation. **c**, *Japonica* subpopulation

GWAS was further conducted using the *indica* and *japonica* subpopulations of the 312 rice accessions and compared with all the identified QTLs (Fig. 3). Since fewer SNPs were used for GWAS in *indica* and *japonica* subpopulations according to the criteria of MAF > 0.05, the *p*-value was reduced for selecting significant SNPs to *p* < 0.001. In total, 7 and 7 QTLs were identified in the *indica* and *japonica* subpopulations, respectively (Table 2). Comparisons of the QTLs identified in different populations indicated that *qCd1, qCd2–1, qCd2–2, qCd4* and *qCd8–1,* which were identified in the whole population, overlapped with *qCd_ind-1*, *qCd_ind-2-1*, *qCd_ind-2-2*, *qCd_ind-4* and *qCd_ind-8*, which were identified in the *indica* subpopulation. The *qCd2–3, qCd3–2, qCd7–1, qCd7–2* identified in the whole population overlapped with *qCd_jap-2*, *qCd_jap-3*, *qCd_jap-7-1* and *qCd_jap-7-2* from the *japonica* subpopulation. The three cloned genes functioning in rice Cd accumulation (*OsHMA3*, *OsNRAMP1*, *OsNRAMP5*) were also located within the interval of *qCd_jap-7-1* and *qCd_jap-7-2*, respectively (Table 2). Interestingly, no common QTL was detected between the *indica* and *japonica* subpopulations (Fig. 3, Table 2).

### Identification of candidate genes related to cd accumulation in grain

**Fig. 4:**
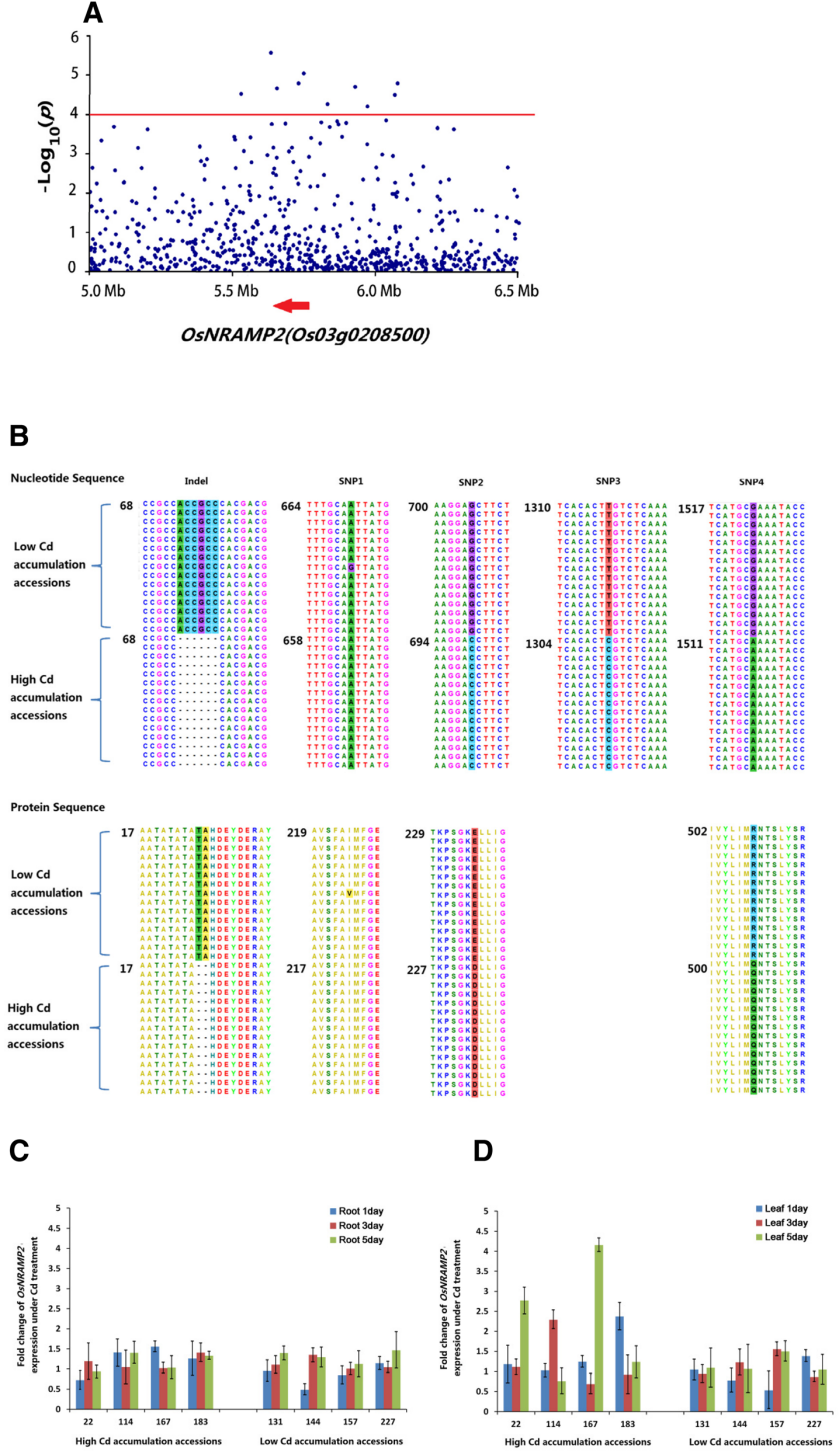
The location of *OsNRAMP2* on chromosome 3 and sequence differences and expression analysis of *OsNRAMP2*. **a**, Colocalization of *OsNRAMP2* with *qCd3–2.* The red arrow indicates the location and direction of *OsNRAMP2*. b, Sequence differences of OsNRAMP2 protein and the respective causal nucleotide differences between high and low Cd accumulation accessions. Sequence differences between high and low Cd accumulation rice accessions are indicated with different background colors. The numbers on the left of each local alignment indicate the position of the first nucleotide/amino acid for the alignment. **c**, Expression changes of *OsNRAMP2* in roots after Cd treatment between high and low Cd accumulation rice accessions. **d**, Expression changes of *OsNRAMP2* in shoot after Cd treatment between high and low Cd accumulation rice accessions

To identify candidate genes related to Cd accumulation in grain, annotations of the genes were analyzed within the identified QTLs (200 kb up and down stream of the most significant SNP of the QTL) based on the Rice Annotation Project (RAP) (Kawahara et al. 2013). Based on its annotation, a potential functional gene (*Os03g0208500*) belongs to the rice NRAMP gene family and is located 14 kb downstream from the most significant SNP in *qCd3–2* (Fig. 4A). The gene symbol synonym of *Os03g0208500* in RAP is *OsNRAMP2*. Since other two members, *OsNRAMP1* and *OsNRAMP5,* have been reported to function in Cd transportation in rice (Sasaki et al. 2012; Takahashi et al. 2011), it was reasoned that *OsNRAMP2* might be the functional gene of *qCd3–2* associated with grain Cd accumulation in rice.

To confirm the function of *OsNRAMP2* in Cd accumulation in rice, the gene structure of *OsNRAMP2* and its expression patterns in both high and low Cd accumulation rice accessions were analyzed. The haplotypes associated with high and low Cd accumulation were determined by analyzing SNPs in the *qCd3–2* region and grain Cd accumulation of the tested rice accessions. In total, 15 rice accessions with low Cd accumulation phenotype and low Cd accumulation haplotype, and 15 accessions with the contrary phenotype and haplotype were selected. The genomic sequences of *OsNRAMP2* in the selected 30 rice accessions were amplified by PCR and individually sequenced. Sequence analysis revealed four SNPs and one Indel in the coding sequence (CDS) of *OsNRAMP2* among the 30 rice accessions (Fig. 4B). Among these differences, there were one Indel and three SNPs between the 15 high Cd accumulation accessions and the 15 low Cd accumulation accessions except for the rice accession 194 from this study. The sequences of allele from low Cd accumulation accessions (designated as *OsNRAMP2-L*) are exactly the same as that from Nipponbare. The allele from high Cd accumulation accessions (designated as *OsNRAMP2-H*) has two amino acid deletions in the 24th and 25th position, as well as an amino acid mutation at the 235th position, with Glu being substituted by Asp, and the 508th amino acid Arg being substituted by Gln. None of these amino acids are within the putative conserved domains previously identified (Cellier et al. 1995; Mani and Sankaranarayanan 2018). However, according to the alignment results with all rice NRAMP genes (Mani and Sankaranarayanan 2018), the Arg in the 508th position is a conserved amino acid across all 7 rice NRAMP genes. This transition may have some functional implications.

To further validate the function of *OsNRAMP2* in rice Cd accumulation, the expression pattern of *OsNRAMP2* in shoot and roots was investigated. The four rice accessions with high Cd accumulation, as well as four accessions with low Cd accumulation, were selected from the 30 rice accessions above. Results demonstrated that the expression levels of *OsNRAMP2* did not significantly change in the roots of any of the eight rice accessions after Cd treatment, compared to their respective controls (*p* > 0.05) (Fig. 4C). However, the expression levels of *OsNRAMP2* in the shoots of the high Cd accumulation rice accessions increased after Cd treatment, while no significant change was observed in low Cd accumulation rice accessions (Fig. 4D).

**Fig. 5:**
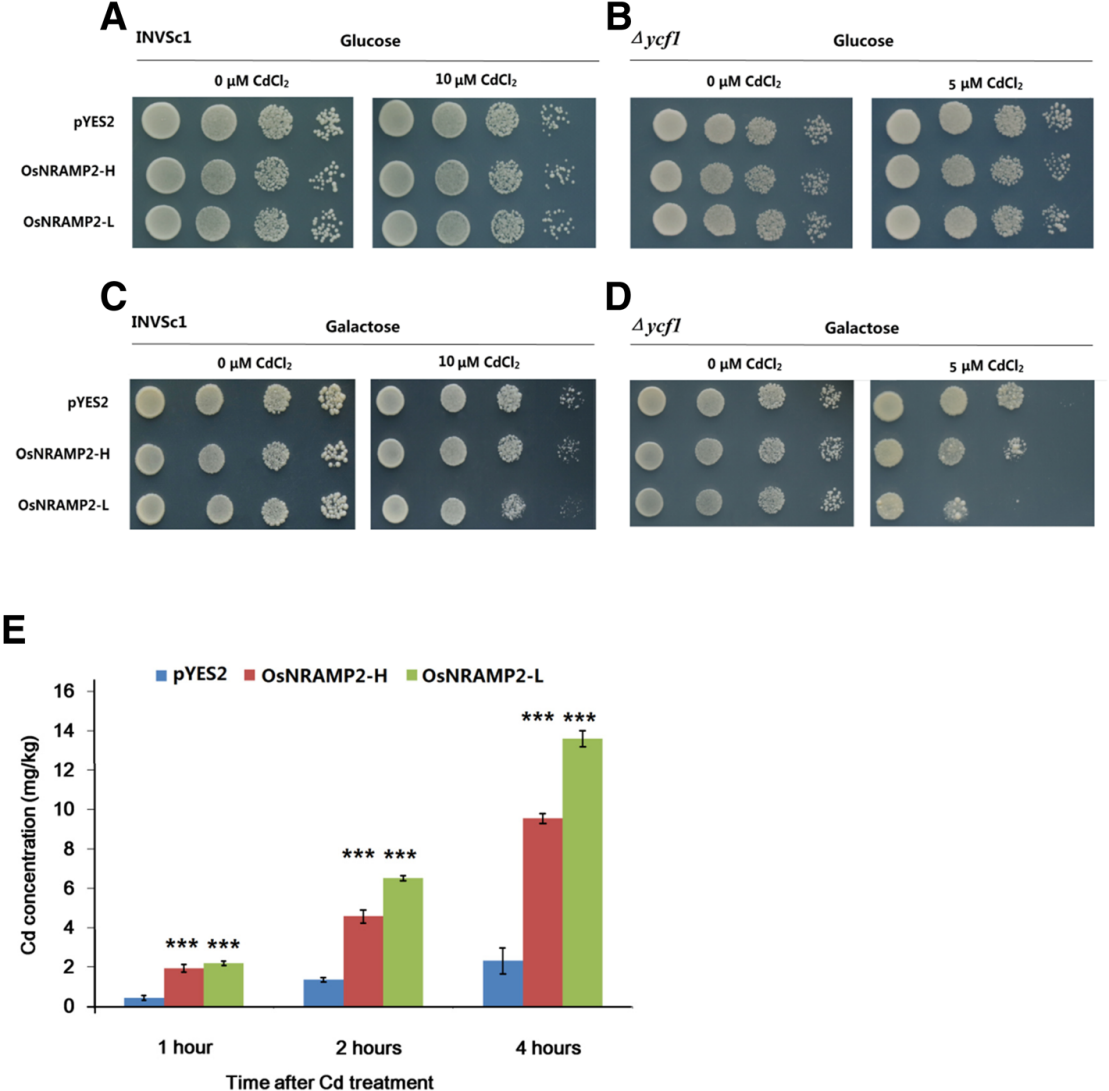
Functional assay of *OsNRAMP2* by heterologous expression in yeast. Yeast INVSc1 strain and *Δycf1* mutant strain expressing the empty vector (pYES2), *OsNRAMP2-H* and *OsNRAMP2-L* were grown in SD-U medium containing concentrations of 0 and 5 μM/10 μM Cd in the presence of glucose (**a**, **b**) to suppress the expression of transformed gene, or galactose (**c**, **d**) to induce the expression of transformed gene. E, Cd accumulation in *Δycf1* strain expressing empty vector and *OsNRAMP2-H*, *OsNRAMP2-L* after exposure to 5 μM Cd for 1, 2 and 4 h. Data are means ± SD of three biological replicates. *** *p* < 0.001 in *t*-test

To investigate Cd transport function of the two alleles of *OsNRAMP2*, expression vectors (pYES2) containing *OsNRAMP2-H* and *OsNRAMP2-L* were used to transform the yeast INVSc1 strain and *Δycf1* mutant strain. The *Δycf1* mutant strain is sensitive to Cd treatment. There was no difference in Cd sensitivity between yeast strains expressing vector control or different alleles of *OsNRAMP2* in the presence of glucose in both the INVSc1 strain and *Δycf1* mutant strain (Fig. 5A, B). In the presence of galactose, which induced gene expression, significant increase in Cd sensitivity was observed in yeast strain *Δycf1* under 5 μM of Cd treatment, and in yeast strain INVSc1 under 10 μM of Cd treatment, when expressing *OsNRAMP2-L*. However, *OsNRAMP2-H* only slightly enhanced yeast Cd sensitivity compared to the empty vector (Fig. 5C, D). Further analysis of the Cd accumulation in *Δycf1* strain expressing different alleles demonstrated that yeast expressing *OsNRAMP2-L* accumulated more Cd than those expressing *OsNRAMP2-H* or the empty vector (Fig. 5E). These results indicate that the OsNRAMP2-L has Cd transport function in yeast, but the Cd transport function of OsNRAMP2-H is reduced due to the change in amino acid sequence.

**Fig. 6:**
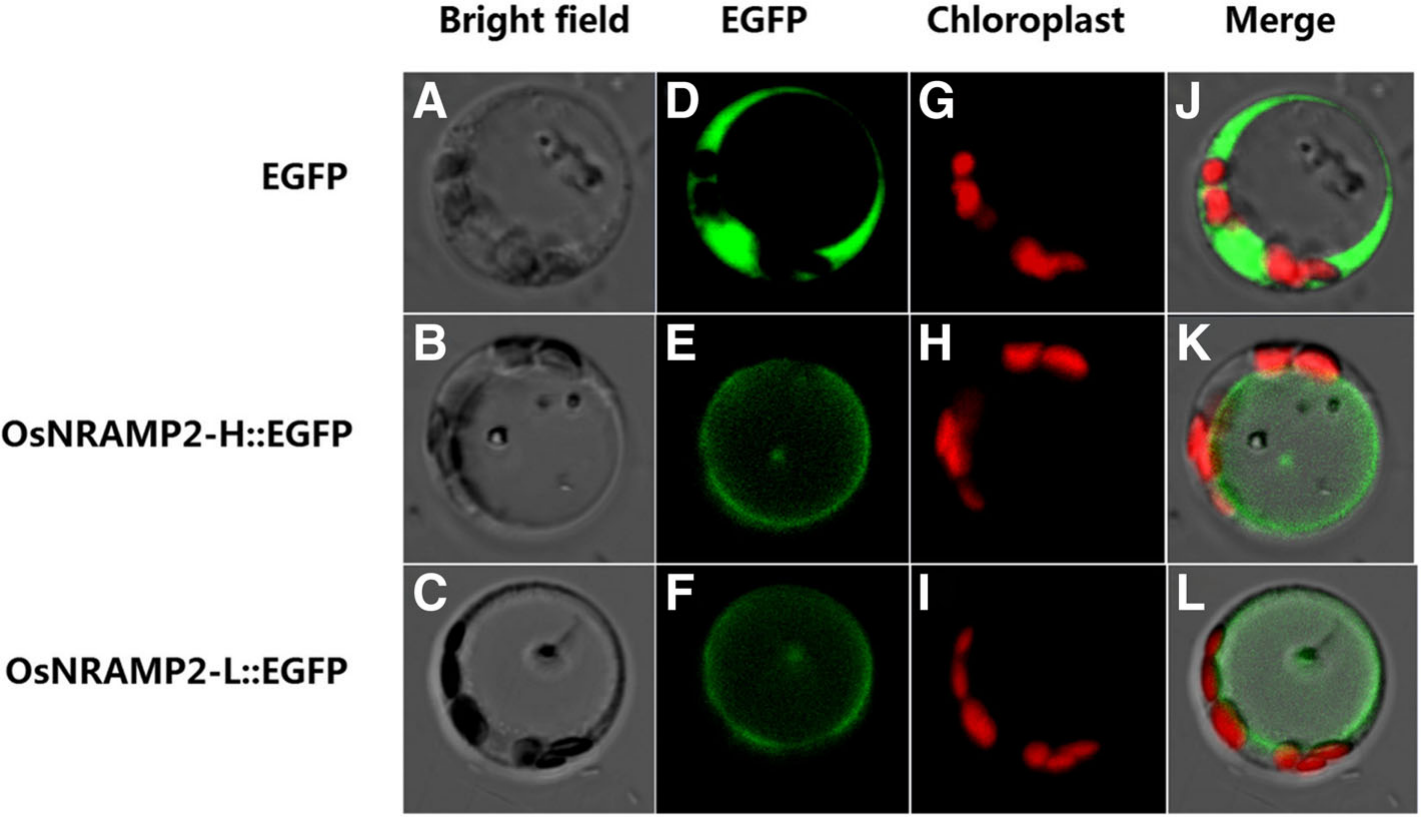
Subcellular localization of OsNRAMP2. Images of bright field (**a**-**c**), EGFP-derived green fluorescence (**d**-**f**), chloroplast auto-fluorescence (**g**-**i**) and merge images (**j**-**l**) of protoplasts expressing EGFP, OsNRAMP2-H::EGFP and OsNRAMP2-L::EGFP

Subcellular localization of OsNRAMP2 protein was further investigated in rice protoplasts. Enhanced green fluorescent protein (EGFP) or OsNRAMP2-L/OsNRAMP2-H fused with EGFP at the C-terminal (designate as OsNRAMP2-L::EGFP and OsNRAMP2-H::EGFP) were transiently expressed in protoplasts from rice seedlings (Zhang et al. 2011). Under confocal microscopy, the green fluorescent signal of both the OsNRAMP2-L::EGFP and OsNRAMP2-H::EGFP was observed outside of the chloroplasts (the chloroplasts showed red signal) (Fig. 6). These results suggest that both proteins are localized to the tonoplast.

## Discussion

### Cd accumulation in grain is diverse in rice germplasm, and is significantly different among subspecies and subgroups

Cd is a highly toxic and mobile element. Consequently, it can easily enter the food chain and become detrimental to human and animal health. To minimize the influx of Cd into the human food chain through rice consumption, Yu et al. (2006) proposed the concept of pollution-safe cultivars (PSCs), i.e. cultivars whose edible parts accumulate a specific pollutant at a level low enough for safe consumption, even when grown in contaminated soil. Selecting or breeding low Cd accumulation cultivars is an attractive and economical method for minimizing the Cd contamination in rice, as the benefit will persist in the seed and reduces the need for other management techniques such as fertilization management or crop rotations.

To search for rice resources with low Cd accumulation for breeding, a diverse rice collection consisting of 312 rice accessions was subjected to evaluation of Cd accumulation. The results showed that Cd concentration in grains among the 312 rice accessions varied considerably, ranging from 0.12 mg/kg to 1.23 mg/kg when these rice accessions were grown in Cd-contaminated paddy fields (Fig. 1A). Similar to previous studies (Arao and Ae 2003, Liu et al. 2005; Pinson et al. 2015; Zhou et al. 2017), the results indicate that the mean grain Cd accumulation in *japonica* accessions is significantly lower than that in *indica* accessions (*p* < 0.001) (Fig. 1B). Furthermore, the mean grain Cd concentration in tropical *japonica* accessions is significantly lower than that in temperate *japonica* accessions (Fig. 1C). This result contradicts previous findings claiming that temperate *japonica* has lower Cd accumulation compared to tropical *japonica* (Yan et al. 2010). Although *indica* cultivars accumulate more Cd, three *indica* accessions with grain Cd concentrations lower than 0.20 mg/kg were still identified. Interestingly, the *indica* “BOL ZO” from the Korean Republic had the lowest grain Cd concentration (0.12 mg/kg) among the 312 rice accessions (Table 1). Together, these results suggest that Cd accumulation in grain is diverse in rice germplasm. However, the Cd accumulation in grain is significant different among subgroups, with accumulation in *indica* > temperate *japonica* > tropical *japonica*. Regardless of subspecies or subgroups, it is possible to screen for rice germplasm with low Cd accumulation, but there is a higher likelihood of identifying them in tropical *japonica*.

### There is a different genetic basis for cd accumulation between *indica* and *japonica* rice

In the present study, 14 QTLs related to Cd accumulation were identified through GWAS using a population consisting of 312 rice accessions. Chromosomal position comparisons reveal that the previously identified genes related to Cd transportation and accumulation, *OsLCD,* O*sHMA3, OsNRAMP1* and *OsNRAMP5*, are located within the QTLs *qCd1*, *qCd7–1* and *qCd7–2* identified in the present study, indicating the reliability of GWAS results in this study.

Comparison of QTLs identified by GWAS in *indica* and *japonica* subpopulations indicates that no common QTL. The three genes, O*sHMA3, OsNRAMP1* and *OsNRAMP5* cloned from *japonica*, were co-localized with *qCd_jap-7-1* and *qCd_jap-7-2* only in the *japonica* subpopulation. It was reported that some *indica* cultivars and temperate *japonica* cultivars contained a loss-of-function allele of *OsHMA3*, in which results in weakened vacuolar sequestration of Cd in the roots and an enhanced Cd accumulation, while many *japonica* cultivars contained a functional allele of *OsHMA3* (Ueno et al. 2010; Yan et al. 2016). Similarly, a significant difference was observed in the expression levels of *OsNRAMP1* between the *indica* and *japonica* subspecies, which may also explain the difference in Cd accumulation between these subspecies (Zhou et al. 2017). Our findings, together with the results from previous studies, suggest that there may be a different genetic basis of Cd accumulation in grain between *indica* and *japonica*, and the genes/QTLs responsible for Cd transportation and accumulation may be subspecies-specific.

Since *indica* rice accumulates more Cd and the cloned genes, so far, are from *japonica* rice, there is an urgent need to identify the genes related to Cd accumulation in *indica* rice and understand their molecular mechanisms. In the present study, three *indica* accessions with very low Cd concentrations in grain (Table 1) and 7 QTLs associated with Cd accumulation in *indica* have been identified by GWAS (Table 2), providing a good basis for the identification of genes responsible for Cd accumulation and molecular breeding for low Cd accumulation in *indica* rice.

### *OsNRAMP2* could be a novel functional gene controlling grain cd accumulation in rice

Through bioinfomatic analysis of the QTLs identified in the present study, *OsNRAMP2* in the *qCd3–2* region was identified. NRAMP is a family of integral membrane proteins identified in many species (Cellier et al. 1995). Previous studies demonstrated that many members of this family could function as metal transporters in yeast and plants (Supek et al. 1996; Thomine et al. 2000). Particularly, various members of this family had been identified as Cd transporters in plants (Clemens and Ma 2016). For example, AtNRAMP6 is a Cd transporter that functions inside the cell either by mobilizing cadmium from its storage compartment or by bringing cadmium to the cellular compartment where it is toxic (Cailliatte et al. 2009).

Altogether, seven *NRAMP* genes have been identified in rice, and five of them (*OsNRAMP1, 3, 4, 5,* and *6*) have been functionally characterized (Mani and Sankaranarayanan 2018). OsNRAMP1 and OsNRAMP5 were identified as Cd transporters in rice. OsNRAMP5 is responsible for the transport of Mn and Cd from the external solution to root cells, but the function of the OsNRAMP1 Cd transporter is not fully understood (Clemens and Ma 2016; Takahashi et al. 2011). However, the function of OsNRAMP2 as a Cd transporter had not been reported previously.

*OsNRAMP2* localizes within a 200 kb region of *qCd3–2* as identified in the present study. There were four amino acid differences in the gene between high and low Cd accumulation accessions. The heterologous assay in yeast (Fig. 5) showed that OsNRAMP2-L can increase Cd sensitivity and accumulation in yeast, while OsNRAMP2-H only slightly increased Cd sensitivity and accumulation in yeast, suggesting that OsNRAMP2 could function as a Cd transporter and OsNRAMP2-L is its functional form. Furthermore, the subcellular localization analysis demonstrated that both OsNRAMP2-L and OsNRAMP2-H are localized at the tonoplast (Fig. 6). Base on previous studies, OsNRAMP1 and OsNRAMP5 are localized to the plasma membrane and mediate metal uptake into the cytosol (Sasaki et al. 2012; Takahashi et al. 2011). In the present study, the OsNRAMP2 protein was localized to the tonoplast, suggesting that it might mediate vacuole-to-cytosol transport of Cd as like AtNRAMP3 and AtNRAMP4, which influence metal accumulation by mobilizing vacuolar metal pool to the cytosol (Lanquar et al. 2005; Thomine et al. 2000; Thomine, et al. 2003). Further physiological, cellular and molecular studies are underway to elucidate the actual function and mechanism of OsNRAMP2 in Cd transport in rice.

## Conclusion

In the present study, Cd accumulation in grain from a diverse rice collection was screened, and QTLs related to rice Cd accumulation were identified through GWAS. The results suggest that the Cd accumulation in grain is significantly different among subgroups (*indica* > temperate *japonica* > tropical *japonica*). Substantial variation also exists within subgroups. It is possible to screen for *indica* germplasm with low Cd accumulation as long as diverse *indica* accessions are investigated, but more success could be found in tropical *japonica*. The fact that no common QTL was identified between the *indica* group and *japonica* group in the present study imply that there may be a different genetic basis for Cd accumulation between *indica* and *japonica*, or that many QTLs for Cd accumulation in rice are subspecies-specific. Through an integrated analysis using GWAS, gene annotation, and functional analysis, it is speculated that OsNRAMP2 could be a functional Cd transporter, and is considered as a novel candidate functional gene associated with Cd accumulation in rice. This study provides new insights into the genetic basis of Cd accumulation, as well as a novel candidate functional gene associated with rice Cd accumulation. These results lay a strong foundation for gene cloning and molecular breeding for low Cd accumulation in rice.

## Methods

### Materials

A total of 312 rice accessions from 53 countries were selected according to the 1568 diverse rice accessions based on their 700,000 SNP genotypes and their origins (McCouch et al. 2016). Seeds of all 312 lines are provided by the International Rice Research Institute (IRRI).

### Field experiment and cd treatment

The field experiments were conducted in 2016 in Renhua, Guangdong, China. The fields are rice-growing paddy fields belonging to light loam soils. Paddy water management, fertilizer applications, and crop protection followed the local farming practices. The 312 rice accessions were planted in a randomized complete design with three replicates. Ten plants per accession were planted in each replicate. The Cd concentration of the field is 1.4 mg/kg on average, which is higher than the second criteria level of the National Environmental Quality Standard for Soil (GB 15618–1995) (People’s Republic of China). The concentrations of other heavy metals (Pb, Cr, Hg, As) are lower than the national standard level 1, indicating that the soil is not polluted by these heavy metals. The pH value of the field was 5.5.

### Sampling and cd detection

To determine the grain Cd concentrations of the 312 rice accessions, 8 plants in the middle of the 10 plants in each replicate were harvested. The grains of eight plants in each replicate were pooled and dried in oven at 70 °C for 24 h, then de-husked. The de-husked grains were grounded into powder and digested with an acid mixture of HNO_3_-HClO_4_. The Cd concentration was determined using a Inductively Coupled Plasma Optical Emission Spectrometry (ICP-OES, iCAP6000,Thermo Scientific, USA).

### GWAS, QTL delimitation and identification of candidate gene

GAPIT version 2 was used for GWAS analysis (Tang et al. 2016). SNPs were selected for GWAS analysis from the 700 K assay of a previous study by the criteria of having less than 15% missing data and minor allele frequency (MAF) > 0.05 (McCouch et al. 2016). GWAS was conducted using the mix liner model with kinship matrix, and PC was set to 2 in GAPIT. Manhattan and QQ plots were produced using R package qqman.

Rice genome sequence version of MSU V7.0 was used as reference for analysis (Kawahara et al. 2013). After GWAS analysis, we follow the criteria of having one associated locus between any two significant SNPs within a 200 kb interval. After determining the QTL of each GWAS analysis, the candidate genes were searched from 200 kb upstream and downstream of the most significant SNP in each QTL.

### Sequencing of *OsNRAMP2* in the rice accessions with low and high cd accumulation

The low and high Cd accumulation rice accessions with 15 accessions each were selected according to the haplotype associated with Cd accumulation within *qCd3–2* and Cd accumulation in the grains of 312 rice accessions. Seedlings of selected rice accessions were used for DNA extraction by the DNeasy Plant Mini Kit (Qiagen, Germany). The DNA samples of the selected rice accessions were used as templates to amplify the full-length genomic sequence of *OsNRAMP2* by KOD-FX polymerase (Toyobo, Japan) using the following primers, forward primer: acaaccactcctagagtccagaga, reverse primer: ggcgacatctcctgaagataacct. After PCR amplification, PCR products were sequenced by Sangon Biotech Co., Ltd. (Shanghai, China). Sequences of *OsNRAMP2* from the 30 accessions were aligned and analyzed by MEGA 7.0 (Kumar et al. 2016).

### Determining the expression patterns of *OsNRAMP2* in rice after cd treatment

Four rice accessions with high Cd accumulation and four rice accessions with low Cd accumulation were selected from the 30 rice accessions mentioned above to analyze the expression patterns of *OsNRAMP2* after Cd treatment. Seeds of selected rice accessions were germinated and grown hydroponically in half-strength Kimura B solution for 2 weeks, with pH adjusted to 5.6 for the duration. The solution was renewed every two days. Plants were grown in an RXZ-1000C growth chamber (Ningbo Jiangnan Instrument Factory, NingBo, China) under a light intensity of 359 μmol m^− 2^ s^− 1^ with a 12 h day length and a relative humidity of 80 ± 5%. The temperature was maintained at 25 °C. After two weeks of growing, plants were treated with 5 μM CdCl_2_ for various days. After Cd treatment for 1d, 3d and 5d, plants were separated into root and shoot, and RNA was extracted by TRIzol (Thermo Fisher, USA) according to the manufacturer’s instructions. Rice plants without Cd treatment were used as control. All assays were conducted in three biological replicates. The RNA samples were reversely transcribed using PrimeScript Reverse Transcriptase (Takara, Japan). Gene expression was determined by qRT-PCR using Biorad CFX96 (Biorad, USA) and SYBR Premix Ex Taq (Takara, Japan).

### Functional analysis of *OsNRAMP2* alleles in yeast

*Saccharomyces cerevisiae* strain INVSc1 (Yan et al. 2016) and Cd-sensitive mutant strain *Δycf1* (Ueno et al. 2010) were used for heterologous expression of *OsNRAMP2-H* and *OsNRAMP2-L*. The ORF of *OsNRAMP2-H* and *OsNRAMP2-L* were amplified by KOD FX (Toyobo, Japan) from the cDNA of high and low Cd accumulation accessions as described above, using the following primers: forward primer, aaaagaattcaaaaaaatgtctatggcgtcgcgcgacctcg, reverse primer, aaaactcgagatcatgtgctctttgtcattgct. PCR products were cloned into expression vector pYES2 (Thermo Fisher, USA). Transformation and selection of empty vector and vectors with *OsNRAMP2* alleles were conducted according to a previous study (Ueno et al. 2010). Positive clones were cultured in SD medium without uracil (SD-U) liquid media with 2% glucose to the early log phase. Five μL of the cell suspension with an initial OD value of 0.1 and three serial 1:10 dilutions were spotted on SD-U plates containing 0 and 10 μM CdCl_2_ for the INVSc1 strain, and 0 and 5 μM CdCl_2_ for the *Δycf1* strain, in the presence of 2% glucose or galactose. The plates were incubated at 30 °C for 3d before the growth phenotypes were evaluated. Cd accumulation in yeast *Δycf1* strains containing pYES2 empty vector and vectors expressing *OsNRAMP2* alleles was determined according to a previous study (Yan et al. 2016). The transformed yeast strains were cultured in SD-U liquid media containing 2% glucose to the early log phase, then enriched by centrifugation and washed with sterile water three times. Cells were then adjusted to the same density (OD600 value 0.2) in SD-U liquid media containing 2% galactose. After incubation for 3 h to induce protein expression, the media was amended with 5 μM CdCl_2_ and the cells were sampled after culturing for 1, 2 and 4 h. The sampled cells were washed with a cold (4 °C) EDTA solution (10 μM, pH 5.0) twice, and then with deionized water twice, and freeze-dried. The cells were weighed, and Cd concentration was determined as described above.

### Subcellular localization of OsNRAMP2 protein

The CDS sequences of *OsNRAMP2-L and OsNRAMP2-H* were amplified using primers as following: forward primer, aaaagaattcatggcgtcgcgcgacctcg, reverse primer, aaaagtcgactgtgctctttgtcattgctgag. The CDS sequences were inserted into the 35S::EGFP vector to produce the OsNRAMP2-L::EGFP and OsNRAMP2-H::EGFP fusion proteins. The fusion plasmids and the empty 35S::EGFP plasmid were transferred into rice protoplasts according to a previous study (Zhang et al. 2011). Laser confocal microscopy (Zeiss, LSM710, Germany) was used to detect fluorescence after incubation for 24 h.

### Data analysis

Phylogenetic tree of all 312 lines was constructed by MEGA 7.0 (Kumar et al. 2016) using SNP data mentioned above. A t-test was conducted using SAS (SAS Institute, 2000) to detect the differences in Cd accumulation in grain between *indica* rice and *japonica* rice, and between tropical *japonica* rice and temperate *japonica* rice.

## Additional files

Additional file 1: **Figure S1.** Phylogenetic tree of the 312 rice accessions based on their genotypes determined by 700 K SNPs (DOCX 279 kb)

Additional file 2: **Table S1.** The Cd accumulation in grain of the 312 rice accession in the present study. (XLSX 34 kb)

## Abbreviations

Cd: Cadmium
CDS: Coding sequence
EGFP: Enhanced green fluorescent protein
GWAS: Genome-wide association study
InDel: Insertion-deletion
MAF: Minor allele frequency
NRAMP: Natural resistance-associated macrophage protein
PSC: Pollution-safe cultivar
QTL: Quantitative trait loci
SNP: Single-nucleotide polymorphism
