# Supplementary material for: Genome-wide association study and candidate gene analysis of rice cadmium accumulation in grain in a diverse rice collection
Source: Rice (N Y). 2018 Nov 21;11:61. doi: 10.1186/s12284-018-0254-x (PMC6249348; doi:10.1186/s12284-018-0254-x)
Supplement: Supplementary file 1 — Figure S1. Phylogenetic tree of the 312 rice accessions based on their genotypes determined by 700 K SNPs (DOCX 279 kb) [file 12284_2018_254_MOESM1_ESM.docx]

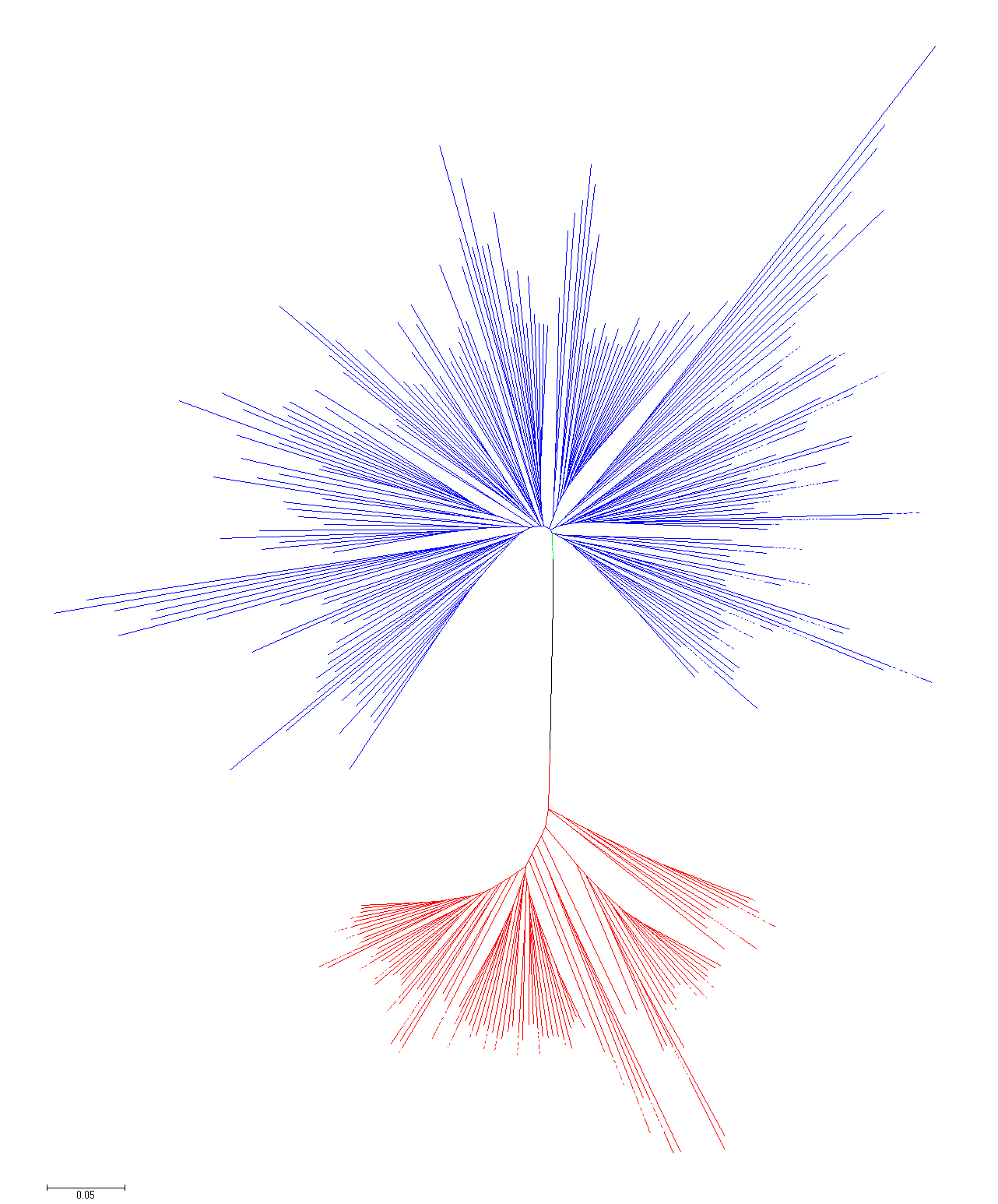


Indica population

Japonica population

Figure S1 Phylogenetic tree of the 312 rice accessions based on their genotypes determined by 700K SNPs
